# Deep learning-based self-induced emotion recognition using EEG

**DOI:** 10.3389/fnins.2022.985709

**Published:** 2022-09-16

**Authors:** Yerim Ji, Suh-Yeon Dong

**Affiliations:** Department of Information Technology Engineering, Sookmyung Women's University, Seoul, South Korea

**Keywords:** self-induced emotion recognition, high-density EEG, channel selection, deep learning, convolutional neural network

## Abstract

Emotion recognition from electroencephalogram (EEG) signals requires accurate and efficient signal processing and feature extraction. Deep learning technology has enabled the automatic extraction of raw EEG signal features that contribute to classifying emotions more accurately. Despite such advances, classification of emotions from EEG signals, especially recorded during recalling specific memories or imagining emotional situations has not yet been investigated. In addition, high-density EEG signal classification using deep neural networks faces challenges, such as high computational complexity, redundant channels, and low accuracy. To address these problems, we evaluate the effects of using a simple channel selection method for classifying self-induced emotions based on deep learning. The experiments demonstrate that selecting key channels based on signal statistics can reduce the computational complexity by 89% without decreasing the classification accuracy. The channel selection method with the highest accuracy was the kurtosis-based method, which achieved accuracies of 79.03% and 79.36% for the valence and arousal scales, respectively. The experimental results show that the proposed framework outperforms conventional methods, even though it uses fewer channels. Our proposed method can be beneficial for the effective use of EEG signals in practical applications.

## 1. Introduction

Emotion plays a crucial role in human decision-making. Hence, recognition of different emotions can effectively improve communication between humans and machines in human-computer interaction (HCI) systems. Human emotions have been recognized using non-physiological signals, such as facial expressions (Ko, 2018), speech (Khalil et al., 2019), and gestures (Noroozi et al., 2018). However, non-physiological signals can be intentionally hidden. In contrast, physiological signals cannot be directly altered because the human body produces them spontaneously. For this reason, many researchers have attempted to identify emotions in physiological signals, such as those detected by electroencephalograms (EEGs), electrocardiograms (ECGs), galvanic skin responses (GSRs), and electromyograms (EMGs) (Wei, 2013; Goshvarpour et al., 2017; Katsigiannis and Ramzan, 2017). In this study, we focus on recognizing emotions using EEG signals.

Previous EEG-based emotion recognition techniques have performed well, but most of them focused on externally induced emotion, using audiovisual materials as emotional stimuli (Koelstra et al., 2011; Soleymani et al., 2011; Zheng and Lu, 2017). This type of method requires subjects to continually pay attention to visual or auditory stimuli. External stimuli may be useful to elicit strong emotions, but because there are individual differences in emotional sensitivity, the selected stimuli may not be suitable for all subjects. Accordingly, some researchers have asked subjects to recall episodic memories or imagine situations associated with certain emotions (Damasio et al., 2000; Onton and Makeig, 2009). This enables the subjects to self-induce emotions based on past experience instead of audiovisual materials determined by researchers in advance. The EEG signals produced by this method are more ecologically valid because they capitalize on individual events that have personal meaning (Salas et al., 2012). However, subjects may lose their concentration when they close their eyes and perform the emotional imagery (EI) task. Therefore, the raw EI signals obtained through this method have a lower amplitude than the signals generated by external stimuli (Iacoviello et al., 2015). This increases the difficulty with which emotions are classified using EEG signals. For this reason, classifying self-induced emotions without using external stimuli remains challenging.

In recent years, deep learning methods have been applied to automatically classify emotions using raw EEG signals without handcrafted features (Craik et al., 2019; Huang et al., 2021). In particular, convolutional neural networks (CNNs) have produced promising results for EEG-based emotion recognition because of their ability to automatically extract robust features (Yang et al., 2018; Hu et al., 2021). However, most CNN-based studies still rely on complex preprocessing techniques, such as the conversion of raw EEG signals into other representations (Kwon et al., 2018; Wang et al., 2020). In this study, we employ a CNN for end-to-end classification, which utilizes raw EEG signals as the input and eliminates the need to perform a complex transformation. Feeding raw EEG signals as input into deep learning models is suitable for analyzing time-series EEG signals (Liang et al., 2021). However, this results in a high computational complexity because of the long training time required when using a large number of EEG channels (Tong et al., 2018). In addition, using all channels, including irrelevant channels, causes the CNN to generate complex features, which decreases the classification accuracy (Wang et al., 2019; Li et al., 2020; Zheng et al., 2021). Consequently, EEG channel selection is advantageous not only for reducing the time required for computation, but also for improving the accuracy.

The most commonly used EEG channel selection methods are the wrapper and filtering methods (Shi et al., 2021). The wrapper method uses recursive techniques to select the optimal subset of all EEG channel combinations (Lal et al., 2004). Wrapper-based methods exhibit superior performance in selecting the optimal channel subset, but they are time-consuming (González et al., 2019) and are prone to overfitting (Alotaiby et al., 2015). Two filtering methods are used to solve this problem. The first involves manually selecting channels related to emotions, and the second automatically selects a subset of channels based on certain standards. For example, many studies have selected EEG channels representing the frontal lobe to capture emotions (Atkinson and Campos, 2016; Thammasan et al., 2016; Xu et al., 2019) because previous results have suggested that the neural activity in the frontal lobe is related to emotional processing. However, manually selecting channels based on previous observations does not necessarily yield better results compared to using all EEG channels. Therefore, this study proposes a statistical method for selecting a smaller number of EEG channels in order to robustly reduce the computational load while simultaneously increasing performance. In this method, the most suitable channels are automatically selected by calculating the EEG signal statistics for each subject before the high-density EEG data are used as input for the CNN.

**Figure 1:**
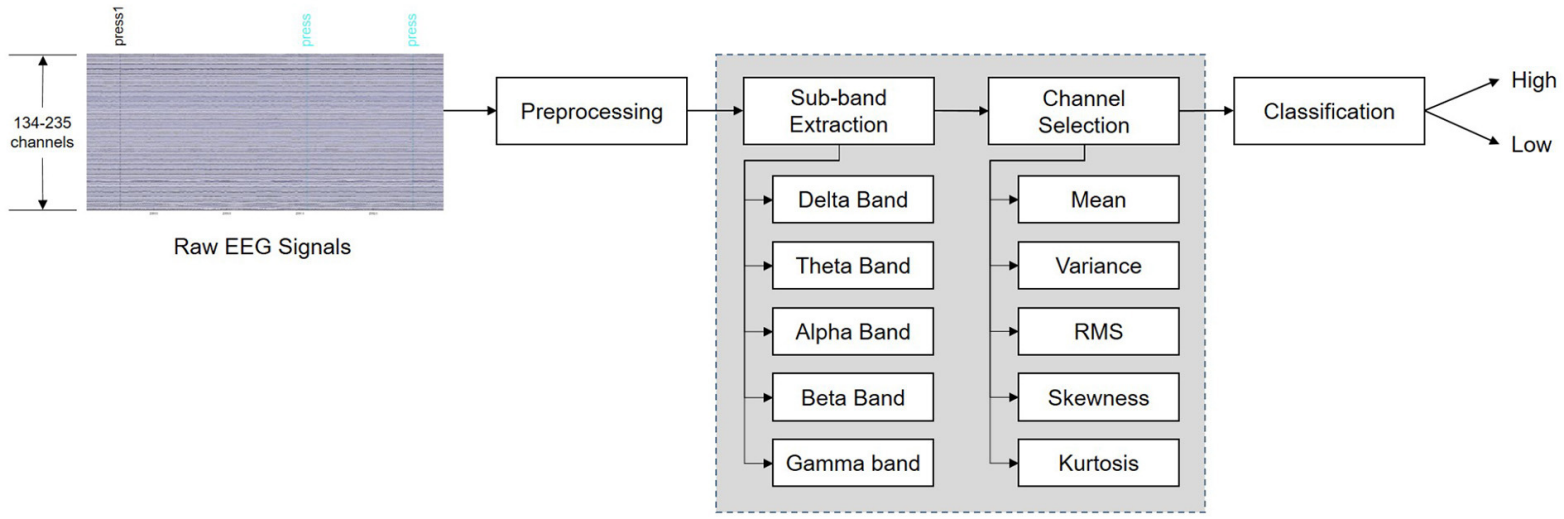
Flow diagram of the proposed system for recognition of self-induced emotions.

In summary, we propose a novel framework for deep learning-based systems using high-density EEG data. In this framework, the optimal frequency band is first selected. Then, after applying a channel selection method using the statistical characteristics of the raw EEG signal data, a CNN is utilized for feature extraction and classification. The flow diagram of the proposed system is shown in Figure 1, and the main contributions of this study are as follows: (1) To the best of our knowledge, this is the first work to classify self-induced emotion in EEG signals using a deep learning model and demonstrate the efficiency of statistical channel selection methods using signal amplitudes; (2) Frequency band and channel selection strategies were applied to pre-select the prominent features of low-amplitude EEG signals to improve the classification accuracy. In particular, a signal statistics-based channel selection strategy that used fewer channels reduced the computational complexity of the system and improved the efficiency of the brain-computer interface (BCI) system, and (3) Experiments were conducted on the publicly available “Imagined Emotion Study” dataset (IESD) to evaluate the performance of our deep learning-based method for classifying self-induced emotion.

## 2. Related work

Many studies have investigated EEG-based emotion recognition, but only a few have classified self-induced emotions using internal EEG signals. For example, Kothe et al. (2013) collected EEG signals of self-induced emotions produced through the recall of experiences associated with 15 different emotions. They used the filter bank common spatial pattern (FBCSP) algorithm to extract temporal-spatial features from 124 channels in the EEG signals and a linear discriminant analysis (LDA) classifier for valence level recognition. They reported an average accuracy of 71.3%, but excluded three ambiguous emotions (compassion, disgust, and excitement). Similarly, Bigirimana et al. (2020) used common spatial pattern (CSP) features to extract the temporal-spatial-frequency representations. They obtained an accuracy of 80% using LDA for imagery induced by recalling sad and happy events. Iacoviello et al. (2015) proposed an automatic real-time classification method based on a discrete wavelet transform (DWT) that used a support vector machine (SVM). They achieved an accuracy of 90.2% for the emotion of disgust self-induced by remembering an unpleasant odor.

Previous studies on self-induced emotions found that emotion-inducing imagery tasks designed to elicit specific discrete emotions (e.g., disgust) achieved higher performance than other methods; however, emotions do not usually occur in isolation (Mills and D'Mello, 2014). To consider emotions similar to those that occur in real life, more studies are needed to classify complex emotions that are mixed with previously experienced emotions. This can be accomplished by including a variety of emotions in the imagery task. Therefore, in this study, we aimed to recognize various self-induced emotions at the valence and arousal levels. In addition, all existing studies on self-induced emotion are based on machine learning (ML) methods. In contrast to these studies, we propose a deep learning-based system to improve the recognition performance and system efficiency. Deep learning methods outperform traditional ML methods in several fields of research (Craik et al., 2019; Roy et al., 2019), but deep learning techniques have not been fully utilized in the classification of self-induced emotion. To the best of our knowledge, this is the first attempt to detect self-induced emotion in EEG signals using a CNN.

## 3. Data description

The EEG dataset we used for training and testing was the “Imagined Emotion Study” dataset (IESD) (Onton and Makeig, 2021), which is publicly available on the OpenNeuro.org platform. To the best of our knowledge, this is the only publicly available dataset that contains EEG signals collected for emotion-inducing imagery tasks. In this dataset, all 34 subjects (with ages ranging from 18 to 35 years) listened to 15- to 30-s audio clips that induced an emotional experience, which helped them imagine what they had felt in the past. Next, the subjects performed EI for an average of 3–5 min for each trial. The EI trials consisted of 15 self-paced emotional images that reflected the emotions of anger, awe, compassion, contentedness, disgust, excitement, fear, frustration, grief, happiness, jealousy, joy, love, relief, and sadness. While the subjects imagined the emotional experience, they pressed the “feeling it” button when they felt the suggested emotion strongly enough. Among the 34 subjects, five were excluded from future analysis because they pressed the “feeling it” button only once per emotion or did not press the button at all. The EEG signals for each subject were collected using a 250-channel BioSemi ActiveTwo system (Amsterdam, Netherlands) with a sampling rate of 256 Hz.

## 4. Preprocessing

### 4.1. Data processing

The raw EEG signals were preprocessed using MATLAB (R2021a, MathWorks Inc., Natick, MA, USA) and its EEGLAB toolbox (EEGLAB, Boston, MA, USA) (Delorme and Makeig, 2004). Four channels (E3, G23, H25, and H26) were not used in this study at all because they were bad channels for all subjects (the E3 and G23 channels were located in the right and left temporal regions, respectively, and the H25 and H26 channels were located in the prefrontal region). Thus, the number of all available channels (*C*) was 246. Furthermore, the data produced by electrodes with poor skin contact were removed from the recorded signals, leaving 134–235 channels per participant (the number of channels differed for each subject because different selections of bad channels were removed for different subjects). Subsequently, artifacts were eliminated by performing independent component analysis (ICA). After the channel subset for each subject was determined, we interpolated across the channels by applying a spherical spline interpolation (Perrin et al., 1989).

In this study, we only used the periods during which the subjects felt the 15 emotions listed in Section 3. We did this because most of the EI trial period covered neutral states that were not related to emotions (Damasio et al., 2000), and thus including the entire period for training could have led to incorrect classification results. Taking this into account, the continuous EEG signals were preprocessed by excluding periods that did not contain data produced by EI. This generated 2-s segments centered on the moment when the subjects pressed the “feeling it” button. Therefore, the number of segments linked to each subject was the same as the number of times the subject pressed the “feeling it” button; this number ranged from 16 to 149 for each subject. The total number of segments used in our study was 1,134.

### 4.2. Label processing

Each segment was associated with a label grouped according to the valence and arousal scales, which are the emotional states quantified using Russell's circumplex model (Russell, 1980). Low valence (LV) indicates “negative” emotions (anger, jealousy, disgust, etc.), and high valence (HV) indicates “positive” emotions (love, joy, happiness, etc.). Low arousal (LA) indicates “calm” emotions (sadness, contentedness, grief, etc.), and high arousal (HA) indicates “active” emotions (excitement, fear, anger, etc.). Low and high values were assigned as 0 and 1, respectively. The labeling results are summarized in Table 1. On both the valence and arousal scales, the subjects felt the emotions belonging to the “high” class more easily, which resulted in more samples being generated for that class.

**Table 1:** Number of samples for each class of emotion.

**Classification scheme**	**Class**	**Number of samples**
Valence	Low (Negative)	498
	High (Positive)	636
Arousal	Low (Calm)	489
	High (Active)	645

## 5. Methods

In this section, we present a novel method that quickly recognizes self-induced emotions in EEG signals. It employs a simple technique that selects frequency bands and channels suitable for classification, and therefore it is computationally efficient and suitable for real-time recognition of emotions

### 5.1. Problem formulation

Let $D_{i}=\left(X^{1},y^{1}\right),\ldots ,\left(X^{N_{i}},y^{N_{i}}\right)$ denote the dataset and *N*_*i*_ denote the number of segments for subject *i*. Given an EEG input for the *k*-th segment, *X*^*k*^, the task is to predict the emotion label *y*^*k*^ corresponding to the *k*-th segment. The input segment *X*^*k*^ of the network is the tensor (*P* × *C* × *N*_*i*_), where P denotes the total number of data points in each segment and *C* denotes the number of EEG channels. Furthermore, *P* = *F*_*s*_ × *T*_*s*_ where *F*_*s*_ denotes the sampling frequency and *T*_*s*_ denotes the duration of the segment. In this context, this study proposes a channel selection method that reduces the number of necessary channels from *C* (all available channels) to *K* without compromising performance.

### 5.2. Frequency band selection

EEG signals are typically categorized according to rhythmic characteristics, resulting in five different sub-bands: delta (δ), theta (θ), alpha (α), beta (β), and gamma (γ). In this study, the EEG signals were band-pass filtered by applying a Butterworth filter to each frequency band. The extracted frequency bands included 1–4 Hz (δ), 4–8 Hz (θ), 8–14 Hz (α), 14–30 Hz (β), 30–50 Hz (γ), and a combination of all these bands. In general, previous EEG-based studies that externally induced emotions using dynamic stimuli, such as video clips, have reported that high-frequency bands are suitable for classifying emotions (Zheng and Lu, 2015; Song et al., 2018; Islam et al., 2021; Rahman et al., 2021). Similarly, the γ band is known to have more of a connection to emotional states than other frequency bands, especially for static stimuli such as images (Li and Lu, 2009; Yang et al., 2020). Accordingly, we hypothesized that the γ rhythm will exhibit a larger difference with different emotions compared to other bands.

However, self-induced emotions evoked by imagining emotional situations in a static environment differ from externally induced emotions. Because the optimal frequency bands for classifying self-induced emotions have not been sufficiently investigated, we investigated all sub-bands in an effort to find a suitable frequency band that maximizes the classification performance.

### 5.3. Channel selection

Channel selection removes irrelevant channels; this task simultaneously reduces the calculation complexity and improves the classification accuracy. An automatic channel selection method has not been developed in the field of emotion recognition, and studies in this field are mainly focused on manually selecting channels based on experience (Xu et al., 2019). A simple method for automatically selecting channels is to use the amplitude statistics of EEG signals as a threshold (Alotaiby et al., 2015). This selection criterion is based on the fact that brain activity is most intense when emotional states are being experienced.

To select channels suitable for classifying self-induced emotions, we considered the typical statistics used in the literature, such as the time-domain statistical values (mean, variance, skewness, and kurtosis) and root mean square (RMS), which can be derived from EEG time series. The variance (standard deviation) has been used for channel selection in epileptic seizure (Duun-Henriksen et al., 2012) and motor imagery classification (Azalan et al., 2019). However, appropriate statistics for channel selection in EI classification have not been reported. Therefore, we propose optimal statistics for classifying self-induced emotions based on the experiments we conducted.

Table 2 presents the mathematical formulation of the statistics used in this study. In these equations, *x*_*c*_(*i*) is the i-th data point of the EEG signal for channel *c* and *N* denotes the total number of data points. The signal statistics were calculated for all channels, and the channels with the highest statistical values were chosen in the channel selection algorithm. Finally, the top *K* channels with the highest classification accuracies were selected.

**Table 2:** EEG signal statistics used for channel selection.

**Statistic**	**Equation**
Mean	$\mu \left(c\right)=\frac{1}{N}\sum_{i=1}^{N}x_{c}\left(i\right)$
Variance	$V\left(c\right)=\frac{1}{N}\sum_{i=1}^{N}{\left(x_{c}\left(i\right)-\mu_{c}\right)}^{2}$
Root mean square	$RMS\left(c\right)=\sqrt{\frac{\sum_{i=1}^{N}|x_{c}\left(i\right){|}^{2}}{N}}$
Skewness	$SV\left(c\right)=\frac{1}{N}\sum_{i=1}^{N}{\left(\frac{x_{c}\left(i\right)-\bar{x_{c}}}{\sigma }\right)}^{3}$
Kurtosis	$KV\left(c\right)=\frac{1}{N}\sum_{i=1}^{N}{\left(\frac{x_{c}\left(i\right)-\bar{x_{c}}}{\sigma }\right)}^{4}$

In the equations, *x*_*c*_(*i*) is the *i*-th data point of the EEG signal for channel *c* and *N* denotes the total number of data points.

### 5.4. Convolutional neural network

After the frequency bands and channels were selected, a CNN automatically extracted features from both the temporal and spatial dimensions of the raw EEG segments. The CNN architecture used in this study 8was based on the shallow CNN (ShallowConvNet) proposed in Schirrmeister et al. (2017). Due to the shallow architecture of ShallowConvNet, a high accuracy can be achieved without significantly increasing the computational cost (Schirrmeister et al., 2017). The architecture of ShallowConvNet is presented in Table 3.

**Table 3:** Architecture of ShallowConvNet.

**Layer**	**Operation and parameters**
L1	40 × Conv(3 × 1), stride(1 × 1)
	40 × Conv(1 × *C*), stride(1 × 1)
	BatchNorm
	Activation(Square)
	AvgPool(30 × 1), stride(4 × 1)
	Activation(Log)
	Dropout(0.5)
Output	Dense
	Softmax classification

The first convolutional layer was split into two layers, performing temporal and spatial convolutions. This was performed because splitting the first convolutional block is known to yield better results when the number of channels is large (Schirrmeister et al., 2017). Hence, this setup is suitable for extracting features from high-density raw EEG signals. Temporal convolution learns how the amplitude changes over time for all channels of the input segment. Because temporal convolution performs computations for all channels, the volume of computations inevitably depends on the number of channels *C*. Therefore, *C* was reduced to *K* through the channel selection method proposed in Section 5.3. Spatial convolution was used to extract the spatial features of each temporal filter. These steps are similar to the band-pass and common spatial patterns (CSP) spatial filter functions in FBCSP (Ang et al., 2008).

The initial convolutional layer was followed by squaring nonlinearity, an average pooling layer, and a logarithmic activation function. These steps are similar to the trial log-variance computations in FBCSP. In the last output layer, the dense and softmax layers were used for classification.

## 6. Experimental results

### 6.1. Implementation details

In this section, we evaluate our proposed method for the task of classifying self-induced emotions in the IESD dataset, using a CNN as the feature extractor and classifier. As mentioned in Section 3, the EEG data for 29 subjects (subject numbers 1–8, 10–21, 23–27, and 29–32) out of a total of 34 were utilized in our experiment. Continuous EEG data were processed into 2-s EEG segments, as described in Section 4.1, and fed as input to the CNN for training and testing. For each subject, 80% of the 2-s EEG segments were used for the training set and 20% were used for the test set. The average values from all fold results using five-fold cross-validation were calculated. Next, we experimentally set the appropriate hyperparameters for ShallowConvNet. The optimized hyperparameters used in this study are listed in Table 4. The experiment was performed on a computer with an Intel(R) Core(TM) i7-10700K CPU @ 3.80 GHz 3.79 GHz and NVIDIA GeForce RTX 3080 graphics processing unit (GPU).

**Table 4:** Hyperparameter values of ShallowConvNet.

**Hyper-parameter**	**Value**
Optimizer	Adam
Learning rate	0.000625
Batch size	8
Epochs	150 [valence]
	50 [arousal]
Loss function	Negative log likelihood

### 6.2. Effect of frequency band on classification performance

In the first set of experiments, the influence of the frequency band on the classification accuracy of the CNN was investigated. Prior to channel selection and feature extraction, all 246 channels were used to find sub-bands suitable for classifying the self-induced emotions. ShallowConvNet was trained separately for the EEG rhythms of the δ, θ, α, β, and γ bands, as well as the entire frequency range of all these sub-bands (1-50 Hz). The average classification results for the 29 subjects on the valence and arousal scales for each sub-band and for all bands using all the channels are shown in Table 5.

**Table 5:** Average classification performance for different frequency bands using all channels.

**Frequency band**	**Valence**		**Arousal**	
	**Accuracy (%)**	**F1 (%)**	**Accuracy (%)**	**F1 (%)**
δ band	62.07	56.33	60.81	56.45
θ band	62.80	57.70	60.32	56.43
α band	64.67	59.62	65.30	61.61
β band	73.39	70.60	71.76	69.16
**γ** **band**	**75.97**	**73.28**	**77.68**	**75.54**
All (δ, θ, α, β, γ)	72.37	68.93	71.24	68.87

The best results are in bold.

Among the five EEG frequency bands, the γ and β bands achieved higher valence and arousal classification results than did the other frequency bands. This result indicates that the higher frequency bands are more closely associated with valence and arousal than the lower frequency bands. The γ band achieved recognition accuracies of 75.97 and 77.68% on the valence and arousal scales, respectively; these were the highest recognition accuracies for each scale. We also considered the F1 score, which is a class-balanced measure of accuracy. Compared to the F1 score of the lowest frequency band (δ), the F1 score of the γ band increased by 16.95% on the valence scale and by 19.09% on the arousal scale. This indicates that the input signals filtered in the γ band (30–50 Hz) improve the precision and recall of the system. In addition, a high average recognition accuracy was achieved for all bands (1–50 Hz). In summary, the CNN performed the best when learning the features in the 30–50 Hz frequency range (the γ band).

### 6.3. Performance comparison of different channel selection methods

**Figure 2:**
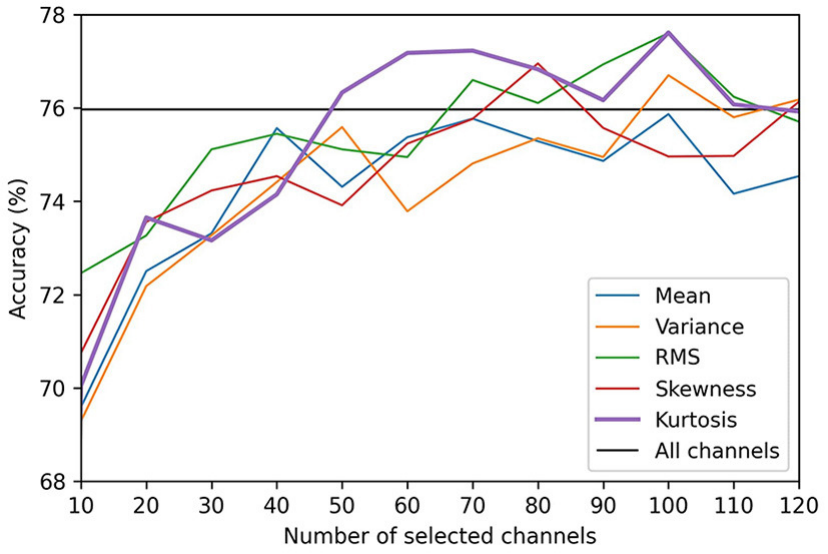
Comparison of valence classification accuracies for different EEG channel selection methods.

**Figure 3:**
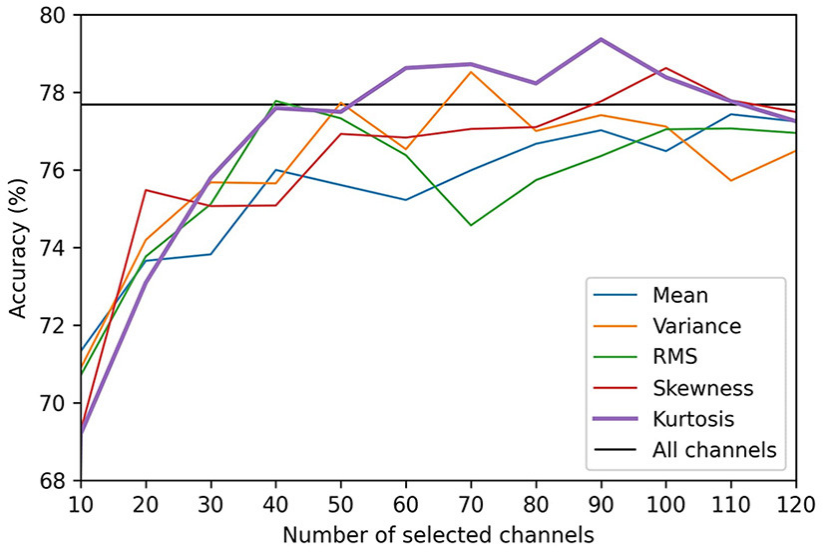
Comparison of arousal classification accuracies for different EEG channel selection methods.

Before comparing the results of the channel selection methods, we first evaluated the influence of the number of selected channels (*K*) on the performance of the CNN. The results produced by varying K from 1 to 123 (half the total number of channels) for the valence and arousal scales are presented in Figures 2, 3, respectively. We did not evaluate the channel selection method using more than 124 channels because, in that case, the channel selection had no significant effect on the results. When K was too small (e.g., *K* = 10), the representation could not be maintained. This led to a decrease in decoding performance, which degraded the accuracy of self-induced emotion recognition. However, when K was too large, similar channels that did not contribute to the classification were also included, which limited the representation capacity of the CNN. Moreover, although there was a minimal improvement in performance, the computational cost of the model significantly increased. In Figures 2, 3, the black horizontal line indicates the accuracy that was achieved when all the channels were considered. On both scales, the accuracy of the kurtosis-based channel selection method began to stabilize after 50 channels. Therefore, in order to determine the optimal number of channels, it is necessary to include more than 50 channels.

Table 6 shows the performance of all the channel selection methods for the γ band. For the kurtosis-based channel selection method, the self-induced emotion recognition accuracy reached 79.03% for the valence scale using the top 68 channels and 79.36% for the arousal scale using the top 90 channels. For arousal classification, the skewness-based channel selection method achieved the highest accuracy (but only marginally) using the top 119 channels. Overall, therefore, the kurtosis-based channel selection method performed the best considering the low number of channels it used.

**Table 6:** Comparison of the accuracy (%) of different channel selection methods for the γ band.

**Classification**	**Statistic**	**Maximum accuracy**	***K* = 64**
**scheme**		**(*K*)**	
Valence	Mean	77.13	74.23
		(79)	
	Variance	77.28	75.06
		(108)	
	RMS	78.15	75.22
		(87)	
	Skewness	76.97	75.33
		(78)	
	**Kurtosis**	**79.03**	**76.50**
		**(68)**	
Arousal	Mean	79.01	75.86
		(122)	
	Variance	78.52	76.38
		(70)	
	RMS	77.78	75.33
		(114)	
	**Skewness**	**79.50**	**76.88**
		(119)	
	**Kurtosis**	**79.36**	**76.80**
		**(90)**	

*K* is the number of selected channels. The best results are in bold.

We also compared the performance of each method using the same number of channels (K=64). This number of channels is commonly used in EEG-based emotion recognition studies. On the valence scale, the kurtosis-based method demonstrated a higher performance than the other statistics. On the arousal scale, the skewness-based method demonstrated the highest accuracy, but it was only 0.08% higher than that of the kurtosis-based method. This illustrates how selecting the minimum number of EEG channels that yields the best or required accuracy can balance the performance and computational complexity (Arvaneh et al., 2011). Therefore, although there was a slight difference in accuracy, the kurtosis-based channel selection method exhibited higher accuracy with fewer channels, and thus it is the most suitable channel selection method for self-induced emotion recognition.

### 6.4. Effect of frequency band on kurtosis-based channel selection

**Figure 4:**
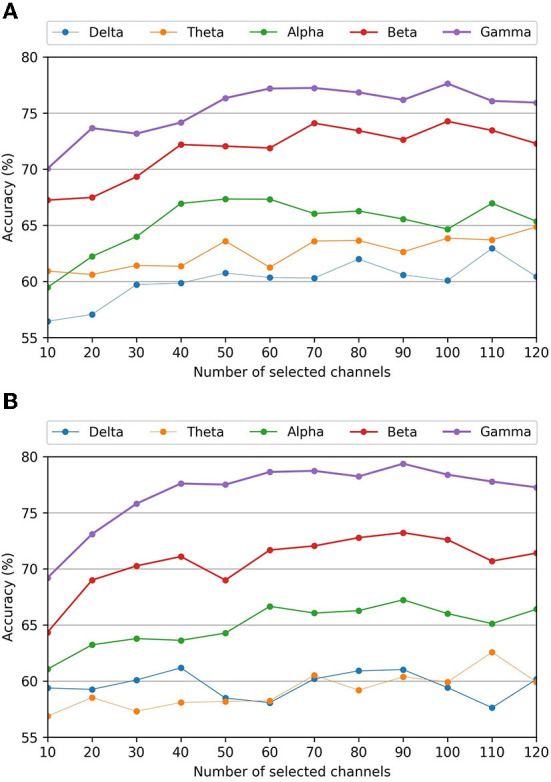
Average classification accuracies of different frequency bands for the kurtosis-based channel selection as a function of the number of selected channels. **(A)** Valence scale classification accuracy. **(B)** Arousal scale classification accuracy.

Figure 4 shows the performance of each frequency band for the kurtosis-based channel selection method. The classification accuracies of the γ band were significantly higher than those of the other frequency bands, regardless of the number of selected channels. In contrast, the classification accuracies of the δ and θ bands were the lowest for the valence and arousal scales, respectively. These results are similar to those obtained using all the channels, as shown in Table 5. This demonstrates that using both the optimal frequency band and optimal channel selection method in our proposed framework improves the EI classification accuracy.

### 6.5. Effect of computational cost reduction

Table 7 presents a comparison of the overall results of the experiments performed in this study. The table displays the average accuracy and standard deviation of the 29 subjects for the valence and arousal classification tasks in terms of the classification accuracy and execution time. The execution time includes the time required for preprocessing, training, and inference, and it represents the overall computational complexity of the system. According to the results, the feature selection (sub-band and channel selection) process significantly reduced the execution time and improved the accuracy. The BPF and channel selection methods were both effective in improving the performance. In particular, the proposed channel selection method exhibited superior performance in terms of reducing the execution time. Here, the channel selection time is less than 0.1-s and accounts for less than 0.01% of the execution time. This confirms that effective channel selection reduces the training time without compromising the accuracy.

**Table 7:** Performance of the proposed framework in terms of average accuracy (%) and execution time.

**Deep learning model**	**Method**	**Valence**		**Arousal**	
		**Accuracy**	**Execution time**	**Accuracy**	**Execution time**
		**(*K*)**	**(for 150 epochs)**	**(*K*)**	**(for 50 epochs)**
ShallowConvNet (Schirrmeister et al., 2017)	Baseline (1-50 Hz)	72.37 ± 15.40	6 m 60 s	71.24 ± 16.11	2 m 20 s
		(246)		(246)	
	BPF (30–50 Hz)	75.97 ± 16.24	6 m 50 s	77.68 ± 13.38	2 m 18 s
		(246)		(246)	
	**BPF** **+** **channel selection (Ours)**	**79.03** **±15.22**	**28 s**	**79.36** **±12.33**	**22 s**
		**(68)**		**(90)**	
DeepConvNet (Schirrmeister et al., 2017)	Baseline (1-50 Hz)	69.67 ± 16.66	30 m 31 s	65.93 ± 15.15	10 m 17 s
		(246)		(246)	
	BPF (30–50 Hz)	73.27 ± 17.63	30 m 02 s	72.89 ± 14.59	10 m 11 s
		(246)		(246)	
	**BPF + channel selection (Ours)**	**75.29** **±15.76**	**5 m 45 s**	**76.10** **±13.98**	**2 m 42 s**
		**(68)**		**(90)**	

BPF stands for “band-pass filter”, which indicates the frequency band selection process. The best results are in bold.

### 6.6. Channel selection results of different model

The advantage of the proposed method is that it does not overfit a specific model. To validate this fact, we applied kurtosis-based channel selection to DeepConvNet (Schirrmeister et al., 2017), which is widely used as a comparison model for ShallowConvNet. Although the optimal set of channels for ShallowConvNet was used as the input for DeepConvNet, the results produced a 79% reduction in execution time without decreasing the accuracy. Thus, the proposed channel selection method can be expected to further improve the accuracy by determining the optimal number of channels for a given CNN.

### 6.7. Subject-independent evaluation

We also conducted experiments on subject-independent evaluation to verify the effectiveness of the proposed method. In this experiment, leave-one-out cross-validation was used for evaluation. In each fold, the EEG data of 28 subjects are used for the training, and the remaining 1 subject's EEG data is used for the testing. Since the data of all subjects except the target subject are used for the training, subject-independent channel selection was performed. Table 8 shows the performance of ShallowConvNet on IESD in 10 epochs. The overall accuracy is lower than that of the subject-dependent experiment. However, after applying BPF and channel selection, performance was improved by the proposed framework. This demonstrates that the proposed method can improve performance in both subject-dependent and subject-independent scenarios.

**Table 8:** Performance of subject-independent classification using ShallowConvNet.

**Method**	**Valence**		**Arousal**	
	**Accuracy**	**Execution**	**Accuracy**	**Execution**
		**time**		**time**
Baseline (1-50 Hz)	59.95 ± 8.96	4 m 10 s	57.71 ± 8.40	4 m 11 s
BPF (30-50 Hz)	62.67 ± 8.57	4 m 08 s	60.29 ± 8.33	4 m 10 s
**BPF + channel selection (Ours)**	**63.46** **±8.34**	**53 s**	**63.75** **±7.11**	**1 m 28 s**

The best results are in bold.

## 7. Discussion

In this study, we automatically classified self-induced emotions *via* a CNN without using complex preprocessing techniques. We demonstrated that the proposed kurtosis-based channel selection method improved the classification accuracy and significantly reduced the computational complexity. In particular, selecting channels from the γ band maximized the overall classification performance.

High-frequency bands have been widely used to study advanced cognitive functions such as emotions (Yang et al., 2020). As a result of evaluating different frequency bands in this study, we also found that the high-frequency bands contributed more significantly to self-induced emotion classification than did the low-frequency bands. In particular, our results demonstrate that the γ band can identify self-induced emotions more clearly than other bands. However, because CNN-based studies have not been conducted for EI classification before, the classification accuracy achieved in this study by ShallowConvNet for each frequency band can be used as a suggestion for future studies.

Statistical channel selection is a classifier-independent (filtering) method. As mentioned in Section 1, filtering methods do not always find the optimal channel subset or improve performance. Despite this fact, the proposed kurtosis-based channel selection method achieved higher performance using fewer channels. We also applied the proposed method to another model (DeepConvNet) to verify the advantages of the filtering method. Although we did not use the optimal channel subset as the input for that model, the computational complexity was significantly reduced without compromising the performance. This is the first study to demonstrate the efficiency of statistical channel selection methods using signal amplitudes, which is based on the observation that self-induced emotions have a lower signal amplitude than those induced by external stimuli.

To the best of our knowledge, no previous study has attempted to classify emotions using the same IESD dataset. In a similar study, Hsu et al. (2022) proposed using unsupervised learning approaches to characterize emotional state changes by clustering emotional states in terms of EEG activity differences rather than using subjective labels within the same dataset. Kothe et al. (2013) used the same experimental paradigm that we used, and their binary classification results for the valence scale produced an accuracy of 71.3%. Therefore, our study outperformed this study in that it yielded a valence classification accuracy of 79.03% using all 15 emotions (as opposed to the 12 emotions Kothe et al., 2013 used) and only 68 channels (as opposed to the 124 channels Kothe et al., 2013 used). Moreover, we achieved an accuracy of 79.36% using 90 channels for the arousal scale, which has not been achieved before in previous studies. Furthermore, the FBCSP algorithm used in the previous study is not suitable for deep learning-based systems because it utilizes multiple sub-bands and incurs high computational costs (Kumar et al., 2017). For this reason, the proposed method is effective in that it selects channels based on amplitude statistics without significant computational demands and reduces the overall computational complexity of the system.

Like other studies, this study has limitations. Based on the fact that the optimal channel subset varies from individual to individual (Almarri et al., 2021), we performed a subject-specific channel selection, but we did not analyze the selected channels themselves. Therefore, our results did not show the relationship between self-induced emotion and selected channels. Further studies need to be done to investigate the relationship between the channels selected by the kurtosis-based channel selection method and channels that are active in the EI tasks. In addition, this study used only EEG signals collected from 29 subjects in the IESD dataset. Therefore, further work will verify our findings and improve classification accuracy by using larger datasets and data augmentation techniques. Furthermore, fusion with other modalities, such as facial expressions, speech, and ECGs, will be considered to improve the classification accuracy.

## 8. Conclusion

This paper presented a new deep learning-based framework for self-induced emotion recognition using high-density EEG signals. We proposed a channel selection method based on signal amplitude statistics to improve the performance by removing irrelevant channels, which avoided the large computational load required by high-density EEG signals. The kurtosis-based channel selection method was the most effective method for maximizing the accuracy of self-induced emotion classification. It achieved average classification accuracies of 79.03 and 79.36% for the valence and arousal scales, respectively, using the IESD dataset. We used only 68 channels for valence scale and 90 channels for arousal scale instead of using all 246 channels in the gamma band. This channel selection method reduced the computational complexity of the system by approximately 89% without causing a decrease in accuracy. In addition, we found that selecting channels from only the γ band generated the highest overall classification accuracy. The experimental results demonstrate that appropriate sub-band and channel selection improve the CNN's ability to learn and extract meaningful features. The selected channel combinations were also applied to other models to evaluate the generalization capability of the channel selection method. This analysis shows that our proposed framework can be applied in future CNN-based emotion recognition studies that use high-density EEG signals. The results of this study may contribute to the efficiency and real-time performance of BCI systems.

## Data availability statement

Publicly available datasets were analyzed in this study. This data can be found at: https://doi.org/10.18112/openneuro.ds003004.v1.1.0.

## Author contributions

YJ designed the methods, performed the experiments, analyzed the results, and wrote the manuscript. S-YD designed the methods, discussed the results, and extensive revisions to the paper. Both authors contributed to the article and approved the submitted version.

## Funding

This work was supported by the National Research Foundation of Korea (NRF) grant funded by the Korea Government (Ministry of Science and ICT, MSIT) (No. NRF-2021R1F1A1052389), by the Commercialization Promotion Agency for R&D Outcomes (COMPA) funded by the Ministry of Science and ICT (MSIT) [Commercialization of health functional foods by verifying the efficacy of functional ingredients and developing the selection method of appropriate content based on AI], and by the MSIT, Korea, under the ICAN (ICT Challenge and Advanced Network of HRD) Program (IITP-2022-RS-2022-00156299) supervised by the IITP (Institute of Information and Communications Technology Planning and Evaluation).

## Conflict of interest

The authors declare that the research was conducted in the absence of any commercial or financial relationships that could be construed as a potential conflict of interest.

## Publisher's note

All claims expressed in this article are solely those of the authors and do not necessarily represent those of their affiliated organizations, or those of the publisher, the editors and the reviewers. Any product that may be evaluated in this article, or claim that may be made by its manufacturer, is not guaranteed or endorsed by the publisher.
